# Quality Control Procedure Based on Partitioning of NMR Time Series

**DOI:** 10.3390/s18030792

**Published:** 2018-03-06

**Authors:** Michał Staniszewski, Agnieszka Skorupa, Łukasz Boguszewicz, Maria Sokół, Andrzej Polański

**Affiliations:** 1 Institute of Informatics, Silesian University of Technology, 44-100 Gliwice, Poland; andrzej.polanski@polsl.pl; 2 Department of Medical Physics, Maria Skłodowska-Curie Memorial Cancer Center and Institute of Oncology, Gliwice Branch, 44-101 Gliwice, Poland; Agnieszka.Skorupa@io.gliwice.pl (A.S.); lboguszewicz@io.gliwice.pl (Ł.B.); Maria.Sokol@io.gliwice.pl (M.S.)

**Keywords:** magnetic resonance spectroscopy, reproducibility, quality control, biomedical signal processing, change points detection

## Abstract

The quality of the magnetic resonance spectroscopy (MRS) depends on the stability of magnetic resonance (MR) system performance and optimal hardware functioning, which ensure adequate levels of signal-to-noise ratios (SNR) as well as good spectral resolution and minimal artifacts in the spectral data. MRS quality control (QC) protocols and methodologies are based on phantom measurements that are repeated regularly. In this work, a signal partitioning algorithm based on a dynamic programming (DP) method for QC assessment of the spectral data is described. The proposed algorithm allows detection of the change points—the abrupt variations in the time series data. The proposed QC method was tested using the simulated and real phantom data. Simulated data were randomly generated time series distorted by white noise. The real data were taken from the phantom quality control studies of the MRS scanner collected for four and a half years and analyzed by LCModel software. Along with the proposed algorithm, performance of various literature methods was evaluated for the predefined number of change points based on the error values calculated by subtracting the mean values calculated for the periods between the change-points from the original data points. The time series were checked using external software, a set of external methods and the proposed tool, and the obtained results were comparable. The application of dynamic programming in the analysis of the phantom MRS data is a novel approach to QC. The obtained results confirm that the presented change-point-detection tool can be used either for independent analysis of MRS time series (or any other) or as a part of quality control.

## 1. Introduction

In localized proton magnetic resonance spectroscopy, (^1^H-MRS), often described as a “virtual biopsy”, the magnetic resonance (MR) signal is acquired from a volume of interest (VOI) prescribed in a human or animal organ. Such in vivo biochemical information defines normal body composition and its perturbation can be monitored by pharmacological and pathological events. Clinical neurospectroscopy provides a direct insight into the metabolic composition of the brain: *N*-acetylaspartate (NAA), the tallest peak in the spectrum, is proportional to the number of functioning neurons, glutamate (Glu) is a neurotransmitter, creatine (Cr) is an energy marker related to mitochondrial function, choline (Cho) is a membrane marker and myoinositol (mI) is a glial marker [1].

^1^H-MRS in vivo is a quantitative technique, yet reliable quantification of absolute metabolite levels is not straightforward. Using an internal standard—for example, the unsuppressed water signal acquired from the same voxel as the water-suppressed one, eliminates several sources of error (such as B1 inhomogeneity, variations in transmitter gain, as well as in hardware performance). Unfortunately, other errors, e.g., uncertainties of water content and unknown relaxation properties still strongly hamper interpretation of the results. Therefore, the metabolite levels are often determined by using a method of independent measurement of a stable phantom with a known content, located in the center of the coil. Such approach is called an absolute standard technique. Reliability of the metabolite levels quantified using this technique depends, inter alia, on the correct adjustment for temporal changes in the scanner performance. Long-term reproducibility monitoring using phantoms is a sensitive method for detection of hardware changes, scanner breakdowns or the results of service engineers’ interventions—all such events directly affect the absolute metabolite levels determined using the absolute standard technique. Usually, the results of such time series measurements are expressed using the coefficients of variance of metabolites [2] or the statistical control charts [3].

MR manufacturers have developed several partially automated methods of MRS data acquisition, aimed at improving reliability and reproducibility of measurements. However, due to scanner differences between various manufacturers, the differences in data collection and analysis methods, and in other technical aspects [4], MRS requires special approaches in order to maintain a high reproducibility of the measurements. The quality control of time series measurements seems to be a crucial component of in vivo MRS data analyses. In medical applications of ^1^H-MRS, the main goal of QC is to establish the performance standards that make it possible to obtain high quality diagnostic data consistent with clinical standards. Thus, the quality assurance and control procedures of the ^1^H-MRS data must be a vital part the performance analysis of MR systems. The clinical and biological laboratories using ^1^H-MRS develop in house QC procedures, which facilitate cross laboratory data comparisons and exchange of data usually being collected within a long time period. Recently, as a part of the QC procedure [5], an unsupervised change-point analysis developed by Taylor [6] has been proposed. There are also many other approaches reported in the literature [7,8,9].

This paper presents a general methodology for QC of the NMR systems based on a long term control of MR systems performed by systematic measurements of the phantom NMR spectra. Uncontrolled variations in the time series of the spectral parameters—called change points [6]—can be detected by a signal partitioning algorithm based on the dynamic programming (DP) algorithm. Application of DP for various tasks related to partition of time series was intensively studied and described [10,11,12]. Here we propose using DP in the quality control in ^1^H-MRS. According to our knowledge it is the first such application of DP available in the literature.

The efficiency of the proposed method was checked by the comparisons of its results with those obtained with the use of other approaches [12]. Both simulated and real phantom spectral data sets were used in the tests. The tested method for QC has been implemented in the tool, which can be downloaded from the dedicated webpage, in the form of Matlab scripts, along with a manual and some additional description of the application and the examples (see Supplementary Materials).

## 2. Materials and Methods

### 2.1. Phantom Data

The phantom NMR spectra used in the study were collected using 1.5T GE scanner (GE Healthcare, Waukesha, WI, USA) from April 2006 to September 2010. A standard head coil and a spherical commercial phantom containing an aqueous solution of the main brain metabolites at physiological concentrations (12.5 mM NAA, 10 mM Cr, 3 mM Cho, 7.5 mM Ins, 12.5 mM Glu, 5 mM lactate, 50 mM KH_2_PO_4_, 12.5 mM NaOH, 0.1% NaN_3_, 1 mL/L magnevist) were applied. The acquisition parameters were: TE (echo time) = 35 and 144 ms, TR (repetition time) = 1500 ms, volume of interest 8 mL, number of signal averages: 64 for water suppressed spectra and 16 for water unsuppressed ones. The time gap between the dates of the measurements (expressed as median) was 8 days (from 2 to 90 days). The dataset includes 118 short TE (35 ms) spectra and 116 long TE spectra (144 ms). However, in the study only 106 short TE spectra and 106 long TE spectra acquired at the same date were used.

The time series analysis was applied to the NAA levels calculated by LCModel—a commercial fitting tool, the current gold standard quantification method [13]. The NAA signal was chosen for the MR system monitoring because the quality control procedures should rely on the most reliable change indicator. *N*-acetyl aspartate shows the largest, singlet peak in the ^1^H-MRS spectra (located at 2.02 ppm); thus, its signal-to-noise ratio (SNR) is the highest. Moreover, as revealed from the phantom studies, NAA is the most stable metabolite in the long period studies [14].

### 2.2. Quantification of ^1^H-MRS Signals

^1^H-MRS data were analyzed using the Linear Combination Model (LCModel) [13]—it comes as a stand-alone commercial software [15] and comprises automated pre-processing to achieve a high degree of user-independence. Its idea relies on the fitting of a basis set (or library) of experimental single metabolite spectra to incorporate maximum information and uniqueness. In our study, the NAA levels were corrected for variable transmitter gain (TG), digital receiver gain (R1) and analogue receiver gain (R2) using the following correction factor:

$fcorr=10^{0.005\cdot \left(TG-65\right)}\cdot 2^{\left(6-\frac{R1}{2}\right)+\left(30-R2\right)}$ and are denoted as NAA_TRA,REC_.

The NAA_TRA,REC_ levels were subjected to time series analysis to identify breakpoints—the points at which changes in the scanner performance occurred. Additionally, water scaled NAA levels (NAA_ws_) were also evaluated.

### 2.3. Partitioning of Time Series

DP algorithm for partitioning of the time series $x_{1},x_{2},\ldots ,x_{N}$ corresponding to the MR phantom metabolite levels was used with the aim of improving quality control of MR system performance.

The concept of DP involves defining *K* blocks, *B*_1_, *B*_2_ ,…, *B_K_* (the data intervals between the change points). The blocks are elements of the time series. The block *B_k_* contains the discrete-time indexes in the range *i, i +* 1, *…, j*. For each block *B_k_* a scoring function *Q*(*B_k_*) is computed, which depends on the metabolite levels *x_k_* measured over the appropriate range *k* ∈ *i, i +* 1, *…, j*. An example scoring function is shown below and it is defined as a sum of the squared differences between a signal and its within-block mean $\bar{x}$,

(1) $$Q\left(B_{k}\right)=Q\left(x_{i},x_{i+1},\ldots ,x_{j}\right)=\overset{j}{\underset{i}{\sum }}{\left(x_{i}-\bar{x}\right)}^{2}$$

Other scoring functions tested are listed in Table 1. A dynamic programming approach can be used for computing an optimal solution and determine the minimal cumulative scoring index:

(2) $$\overset{K}{\underset{k=1}{\sum }}Q\left(B_{k}\right)\to min$$

which is accomplished by recursive application of the following Bellman’s equation:

(3) $$Q_{1\ldots j}^{opt}\left(k+1\right)=\underset{i=1\ldots j-1}{\min}Q_{1\ldots i-1}^{opt}\left(k\right)+Q\left(x_{i},x_{i+1},\ldots ,x_{j}\right)$$

In the above recursion $Q_{1\ldots i}^{opt}\left(k\right)$ denotes optimal, partial, cumulative score resulting from optimal partitioning of the range 1*…i* into *k* blocks. The solution of the optimization problem, $Q_{1\ldots N}^{opt}\left(K\right)$, is given by the result of (3) for *j* = 1, …, *N* and *k* = 1, …, *K*.

**Figure 1:**
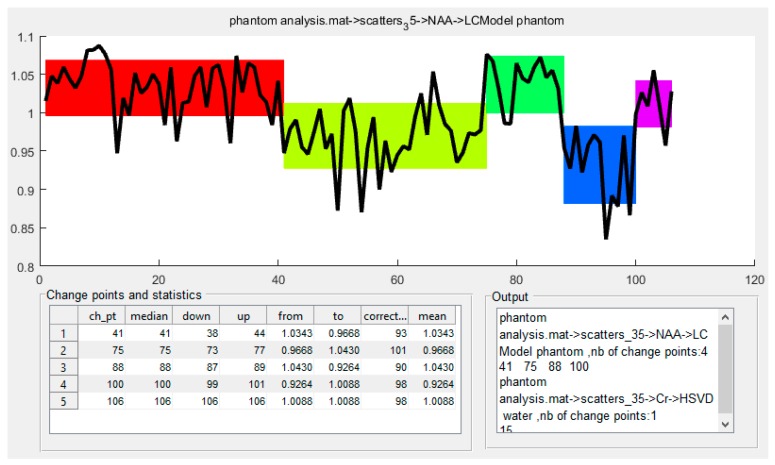
The main window of the time series analysis tool for quality control showing the change points and the statistics results obtained for exemplary data. The color bands correspond to the mean values (± standard deviation) of the signal in the monitored time intervals. The table in the lower-left corner presents: change points (ch_pt), medians, lower and upper confidence intervals (down and up), magnitude of the signal change (from/to) expressed as the mean signals in the time intervals before and after the change point, the correction factor computed as: (mean signal in the time interval after the change point/mean signal in the first interval) × 100%, mean signal in the time period before the change point.

Estimation of the number of change-points constitutes an important issue in the whole procedure and is performed with use of the Bayesian Information Criterion (BIC) [16]. The default implementation of our tool assumes unsupervised estimation of the number of change points based on BIC, but the users can also define their numbers manually via the user-friendly GUI. Figure 1 shows the GUI presenting the results of the analysis of exemplary time series (visualization of temporal variability of the signal and corresponding statistics [17]).

The same time-series data were analyzed using also a set of the methods available as Matlab functions [12]. In the mentioned paper, Arlot et al. present a comprehensive analysis of various change points detection algorithms. Among the methods used are the penalized maximum likelihood (PML) [18], Zhang and Siegmund (ZS) [19], empirical risk minimization (ERM) [20] and classical examples of cross validation methods that are the leave-one-out (LOO) [21] and V-fold cross-validation (VFCV) [22]. These algorithms were applied by us due to their common application of DP which was used to evaluate changes in heteroscedastic data having different variations of the noise in time series. We also tested the Matlab function wvarchg (applied for change points analysis in [23]) and the Change Point Analyzer (CPA) [6].

The scripts were written and tested in Matlab 2013b under macOS (2.3 GHz Intel Core i7, 16 GB RAM). Additionally, the methods were verified in Matlab 2016a under Windows 10 Pro 64-bit Operating System, x64-based procesor (Intel(R) Core(TM) i7-4790 CPU @ 3.60 GHz, RAM 32.0 GB).

## 3. Results

### 3.1. Simulated Data

The DP algorithm was implemented with various scoring functions *Q*(*Bk*) listed in Table 1.

The effectiveness of various algorithms in change-point detection was tested and validated using 100 randomly generated datasets. Each of the datasets (including *N* = 106 data points) corresponded to the hypothetical time change of the MR metabolite level. Additionally, 10 change-points along the time axis, corresponding to the uncontrolled changes of the measurement system parameters were randomly generated. The datasets were distorted by a white Gaussian noise with randomly generated SNR per sample in a given segment (from 1 to 10 dB), ensuring heteroscedasticity of the data. The performance of various methods was evaluated for the predefined number of change points ranging from 1 to 15 based on the error values calculated by taking difference between n original data points (Data) and mean values in interval between change-points (Means) according to the equation:

(4) $$Error=\frac{1}{n}\overset{n}{\underset{i=1}{\sum }}\sqrt{{\left(Data_{i}-Means\right)}^{2}}$$

**Figure 2:**
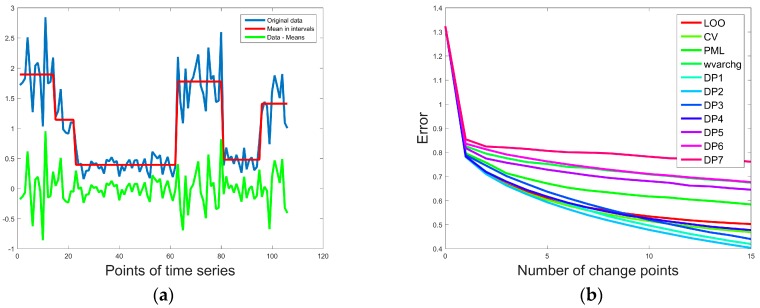
Results of the analysis of random time series using our online quality control (QC) tool: (**a**) marked mean values in the intervals between change-points and residuals computed as differences between original data points and means in the corresponding time intervals; (**b**) relation between the error (computed using Equation (4)) and the number of change points obtained using various methods.

Beside DP with various scoring functions (Table 1) we compared a set of the methods: PML, LOO, CV (in our tests the methods such as ZS, ERM and VFCV gave similar results, so they are grouped in CV in Figure 2b) and Matlab implementation of the function wvarchg. Figure 2a shows the random time series (Original data in blue) with the marked mean values in the intervals between the change-points (Mean value in intervals in red), and the difference between the original data and the interval mean values (Data—Means in green). The relation between the number of change-points and error (computed using Equation (4)) is presented in Figure 2b. According to the obtained results, the DP algorithm with scoring function DP2 (2) reveals the lowest error.

### 3.2. Analysis of Phantom Data

**Figure 3:**
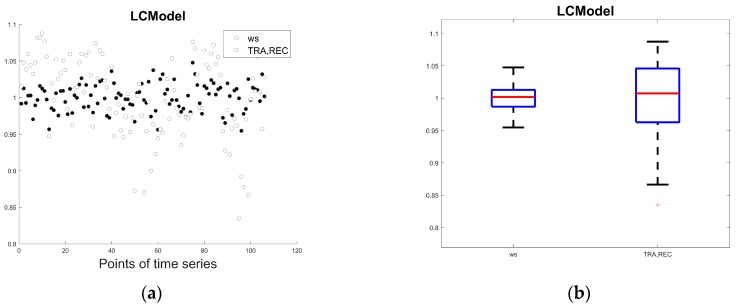
Temporal variability of the water scaled N-acetylaspartate (NAA_ws_) and NAA_TRA,REC_ values obtained from short TE ^1^H-MRS spectra (TE 35 ms): (**a**) scatterplot; (**b**) boxplot.

Figure 3a,b show the comparison of the temporal variability of the NAA_TRA,REC_ and NAA_ws_ levels obtained from the short TE ^1^H-MRS spectra in the period from April 2006 to September 2010. As expected, the NAA_ws_ levels are characterized by a lower temporal variability than NAA_TRA,REC_ due to a compensation of various sources of error by using water reference signal measured from the same voxel as a water suppressed spectrum. Scanner performance instability is seen for the NAA_TRA,REC_ data, indicating a need for additional analysis of the time series.

### 3.3. Analysis of ^1^H-MRS Time Series

**Figure 4:**
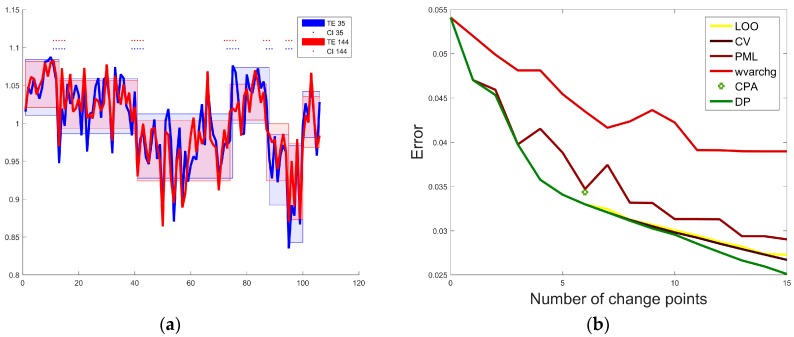
Time series analysis of the NAA_TRA,REC_ levels obtained from the short (blue) and long (red) TE ^1^H-MRS spectra: (**a**) visualization of the change points and corresponding confidence intervals (CI); (**b**) the relation between the error values (computed using Equation (4)) and the change points numbers obtained using various methods for short TE.

Figure 4a presents the results of change point analysis of the NAA_TRA,REC_ levels obtained from the short and long TE ^1^H-MRS spectra using DP method included in our script with the scoring function DP2 (2). It is evident that the change-points and their confidence intervals (CI) are similar for the short and long TE data. Table 2 presents the mean values (mean) and standard deviations (std) for the following spectral parameters: FWHM (full width at half maximum), SNR and TG obtained from the spectra averaged for the time intervals detected by change point analysis. R1 and R2 were equal to 13 and 28, respectively, within the whole time.

The short TE MRS time series were also analyzed using CPA (bootstrap = 1000, mean square error estimator), the methods PML, LOO, CV and Matlab function wvarchg. To compare the performance of these methods, the relationship between the number of change-points (varied from 1 to 15) and the error values (computed using Equation (4)) was investigated.

Since it is not possible to change number of change-points in CPA, the error value for six change points (determined by CPA) was accepted in the comparisons of the techniques. Figure 4b presents the NAA_TRA,REC_ errors obtained for the assessed methods as a function of the change-points number, while Table 3 compares the error values computed according to the Equation (4) for 6 change points using various change-point detection techniques. The comparison reveals that the application of DP, CV and LOO methods results in the lowest error value, regardless the number of change points defined. However, with an increasing number of change points, the DP method shows its superiority. The difference in the QC dates (expressed in median) is 8 days which means that we used data not uniformed over time. However, it does not influence the DP approach because the scoring functions are calculated for the sequential data and the modeling provides the same results.

## 4. Discussion

Although ^1^H-MRS in vivo technique has proved its usefulness in neurodiagnostics, its application is still limited. The metabolite levels obtained from ^1^HMRS spectra are prone to errors resulting from temporal variations in the scanner performance. Therefore, many efforts are being undertaken to develop effective quality control procedures [25]. Both specialized, multicompartmental phantoms and commercial MRS spheres filled with a brain mimicking solution (as used in our study) are exploited in these procedures [4,26,27]. Although long-term monitoring with the use of multicompartmental phantoms provides more detailed technical characterization of the MR system (for example in terms of the signal localization/suppression efficiency, which is of crucial importance in case of spectroscopy), the instabilities detected using commercial phantoms are also sensitive (but not specific) indicators of the changes affecting absolute metabolite levels (such as flip angles, coil tuning, loading correction, gradient calibration, receiver settings, etc.) [28]. The long-term monitoring of MR system for possible disturbances of its work—manifesting itself as the change points—may be helpful in detecting and characterizing the reasons of these disturbances and, thus, also in stabilization and improvement of the MR system quality indexes. As revealed from our long-term MR system observation, the change points may be usually related to the ‘natural breakpoints’ of the system (as the dates of 19 October 2006—system breakdown, 30 October 2007—service engineer interventions, 19 December 2008—software upgrade in our long-time series data). However, sometimes it is difficult to correlate the change points with the critical events in the scanner performance history recorded by service engineers [5]. This underscores the need for independent quality control procedures for MRS spectroscopy and raises the important role of unsupervised techniques of change point detection in these procedures. Although there is an ongoing debate on the phantom design and definition of the parameters to be monitored, the role of change point detection techniques in the quality control of an MR system has not been paid attention so far. Such analysis is crucial for a proper interpretation of large datasets collected during long periods of time [29]. Comparison of the spectra acquired from patients suffering from rare disorders or monitored longitudinally would definitely be facilitated by progress in this field.

## 5. Conclusions

Partitioning of NMR time series oriented towards detection of change-points can be a part of quality control procedures. Our motivation for publishing the scripts and the change-point-detection tool used in this work was to facilitate the monitoring of MR scanner performance over time by applying a completely automated procedure. Such a procedure is able to distinguish between common and special causes of system variations.

Dynamic Programming with different scoring function was applied for change point detection and this approach was tested and validated for a randomly generated time series distorted by white noise. The real data collected for four and a half years was taken from the phantom quality control studies of the MRI scanner. The time series analyses using external software CPA, a set of external methods and the proposed tool show comparable results. The reliable results confirmed that our change-point-detection tool can be used either for the independent analysis of NMR time series (or any other) or as a tool facilitating and visualizing the quality control process.

## Figures and Tables

**Table 1:** Various scoring functions for our implementation of dynamic programming (DP), where σ denotes a standard deviation, $\bar{x}$ is a mean value and n is a number of points in segment.

DP1	DP2 (2)	DP3	DP4
$\sum_{i}^{j}\left|x_{i}-\bar{x}\right|$	$\sum_{i}^{j}{\left(x_{i}-\bar{x}\right)}^{2}$	$\frac{\sum_{i}^{j}{\left(x_{i}-\bar{x}\right)}^{2}}{\bar{x}}$	$\frac{\sum_{i}^{j}{\left(x_{i}-\bar{x}\right)}^{2}}{\sigma }$
DP5	DP6	DP7	
$\frac{\sigma }{\max\left(x_{i}\right)-\min\left(x_{i}\right)}$	$\bar{x_{i}-\bar{x}}$	$\frac{1}{n}\sum_{i}^{j}\sqrt{{\left(x_{i}-\bar{x}\right)}^{2}}$	

**Table 2:** Mean values (mean) and standard deviation (std) of full width at half maximum (FWHM), signal-to-noise ratio (SNR) and transmitter gain (TG) of the spectra averaged for the time intervals detected by the change point analysis. FWHM and SNR (the ratio of the maximum in the spectrum-minus baseline over the analysis window to twice the root mean squares of residuals) are the main spectral quality parameters determined with use of Linear Combination Model (LCModel) [24].

	From 3/4/2006 to 19/10/2006	From 31/10/2006 to 30/10/2007	From 5/12/2007 to 19/12/2008	From 20/12/2008 to 10/12/2009	From 22/12/2009 to 2/2/2010	From 24/2/2010 to 22/6/2010	From 6/7/2010 to 21/9/2010	Whole Time Interval
Change point position	13	41	75	88	95	100		
FWHM [ppm] mean + std	0.0207 0.0039	0.0208 0.0039	0.0216 0.0044	0.0213 0.0044	0.0233 0.0053	0.027 0.0045	0.0219 0.0049	0.0216 0.0044
SNR mean + std	33.8333 3.3799	32.9286 2.3716	31.5 3.0674	32.0769 2.3616	32.8571 2.2678	25 6.5955	34 2.1602	32.1604 3.4430
TG [0.1 dB] mean + std	144.25 1.2154	141.8214 1.7858	141.2058 2.2263	140.9231 1.6053	139.8571 1.7728	142.286 1.6036	142.2857 1.6036	141.604 2.1559

**Table 3:** The error values (computed using Equation (4)) obtained from the NAA_TRA,REC_ levels series analysis for a predefined number of six change points. The time points correspond to the following dates of the measurements: 13—19 October 2006, 41—30 October 2007, 75—19 December 2008, 88—10 December 2009, 95—2 February 2010, 100—22 June 2010.

DP	CV	LOO	PML	wvarchg	CPA
Error for NAA obtained from LCModel for 6 change points					
0.0330	0.0330	0.0330	0.0347	0.0435	0.0344
Position of detected change points					
13; 41; 75; 88; 95; 100	13; 41; 75; 88; 95; 100	13; 41; 75; 88; 95; 100	41; 75; 78; 80; 88; 100	49; 50; 51; 94; 99; 100	12; 41; 60; 75; 88; 100

## Supplementary Materials

The following are available online at http://www.mdpi.com/1424-8220/18/3/792/s1. The presented paper is submitted along with source codes of all scripts and gui written in Matlab (scripts.zip), as well as manual with full explanation of steps required in application of scripts.
