# Supplementary material for: Quality Control Procedure Based on Partitioning of NMR Time Series
Source: Sensors (Basel). 2018 Mar 6;18(3):792. doi: 10.3390/s18030792 (PMC5877107; doi:10.3390/s18030792)
Supplement: Supplementary file 1 [file sensors-18-00792-s001.zip › Supplementary Materials/manual/1 About project.pdf]

Tool for analysis of time series of NMR data

Tool for analysis of time series of NMR data

Medical centres are obliged to store past medical records, including the results of quality assurance (QA) tests of the medical equipment which is especially useful in checking reproducibility of medical devices and procedures.
Analysis of time series is an important part of quality control of NMR data and for that reason the authors have propose the online QA tool in the form of user friendly GUI and Matlab scripts. The presented tool performs change point detection by application of dynamic programming. Its utility was confirmed on randomly generated data and on the set of real phantom data.

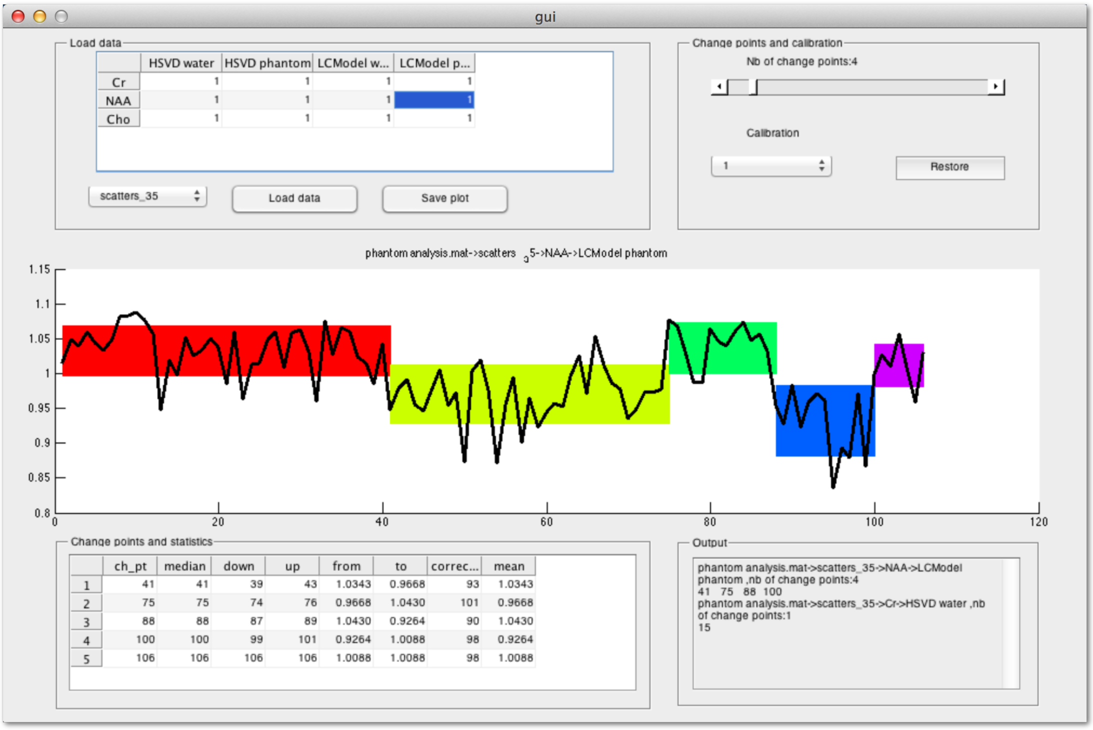

Copyright © All Rights Reserved.
