# Supplementary material for: Quality Control Procedure Based on Partitioning of NMR Time Series
Source: Sensors (Basel). 2018 Mar 6;18(3):792. doi: 10.3390/s18030792 (PMC5877107; doi:10.3390/s18030792)
Supplement: Supplementary file 1 [file sensors-18-00792-s001.zip › Supplementary Materials/manual/2 GUI.pdf]

Tool for analysis of time series of NMR data

GUI

1. Data preparation and loading.
2. Launch GUI.
3. First run.
4. Preview of time series.
5. Choosing data.
6. Load data and save results.
7. Change points detection and calibration.
8. Visualization of results.
9. Statistics of results.
10. History window.
11. Possible problems.

1. Data preparation and loading.

The GUI allows the user to analyze complex datasets and to choose the data storage method. The only requirement is that data have to be stored in a cell array of at least 2x2 dimension (or bigger). The first column and the first row (besides the 1x1 block which should be left empty) are dedicated for the names stored as char:

```
>> name_of_cell{2,1} = 'name of first row'
>> name_of_cell{1,2} = 'name of first column'
```

The time series should be saved as a vector of double type.

```
>> data(number of data points,1)
```

In order to put the data into the cell array it is required to type the following line:

```
>> name_of_cell{2,2} = data
```

In the workspace the user can have many cells and all of them can be saved in .mat file that is accepted by GUI.

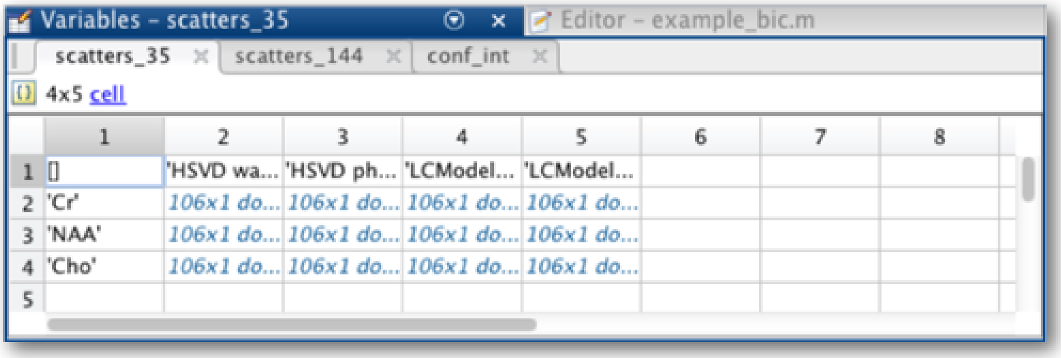

	1	2	3	4	5	6	7	8
1	[]	'HSVD wa...	'HSVD ph...	'LCModel...	'LCModel...			
2	'Cr'	106x1 do...	106x1 do...	106x1 do...	106x1 do...			
3	'NAA'	106x1 do...	106x1 do...	106x1 do...	106x1 do...			
4	'Cho'	106x1 do...	106x1 do...	106x1 do...	106x1 do...			
5								

Alternatively, the user can load the data from a spreadsheet saved in .xls file. The time series should be placed in separate columns.

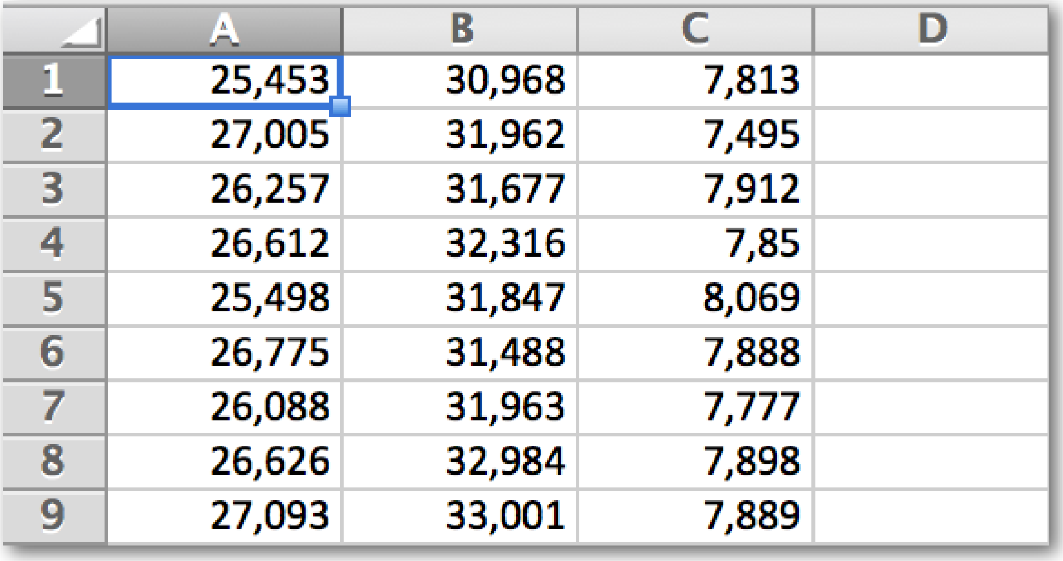

	A	B	C	D
1	25,453	30,968	7,813	
2	27,005	31,962	7,495	
3	26,257	31,677	7,912	
4	26,612	32,316	7,85	
5	25,498	31,847	8,069	
6	26,775	31,488	7,888	
7	26,088	31,963	7,777	
8	26,626	32,984	7,898	
9	27,093	33,001	7,889	

2. Launch GUI.

The GUI can be launched from a Matlab command line by typing gui

```
>> gui
```

3. First run.

When the GUI launches, the first dialog box is opened which enables the user to choose .mat file (prepared according to point 1). The GUI calculates automatically the change points for the chosen dataset (which is name_of_cell{2,2}).

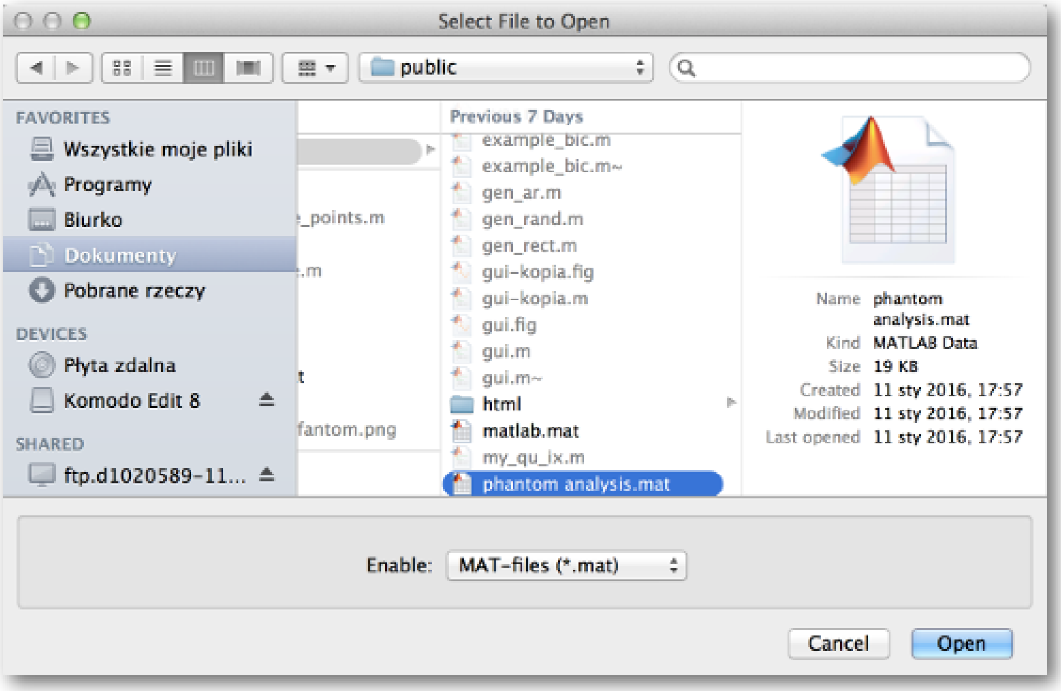

Alternatively, the user can choose and load the data from a spreadsheet saved in .xls file.

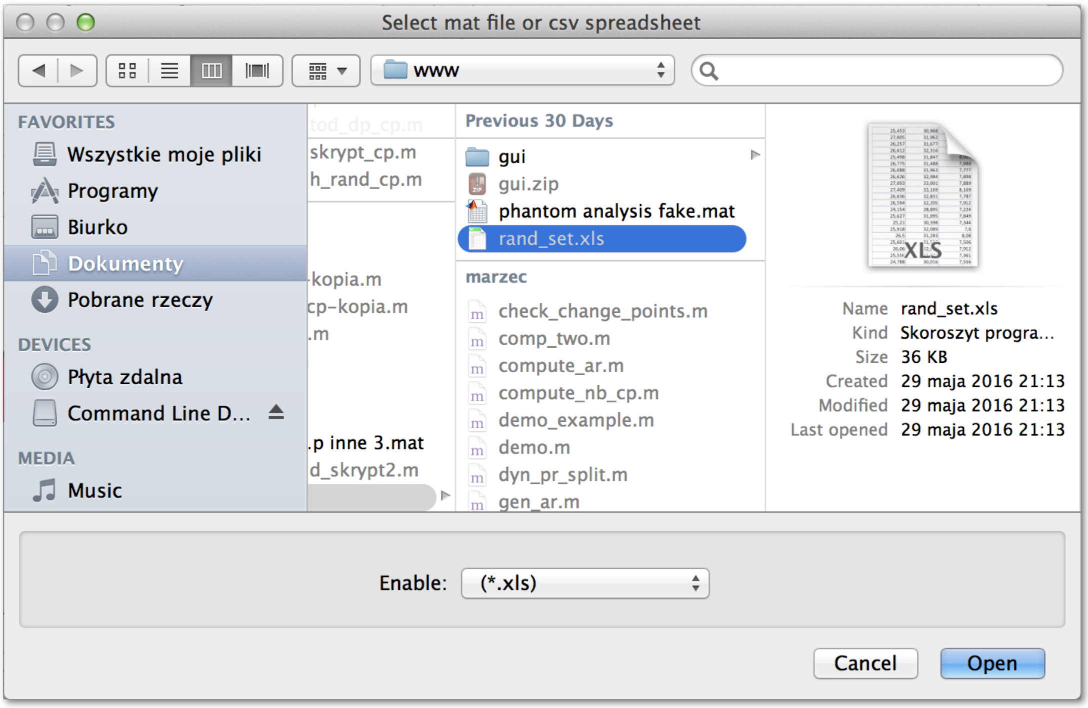

4. The preview of time series.

The preview of the cell array content stored in .mat file is visible in the table located in the upper-left corner of the GUI. The user can choose time series by clicking corresponding '1' in the cell array. After that the GUI automatically calculates the change points and displays the results graphically and numerically.

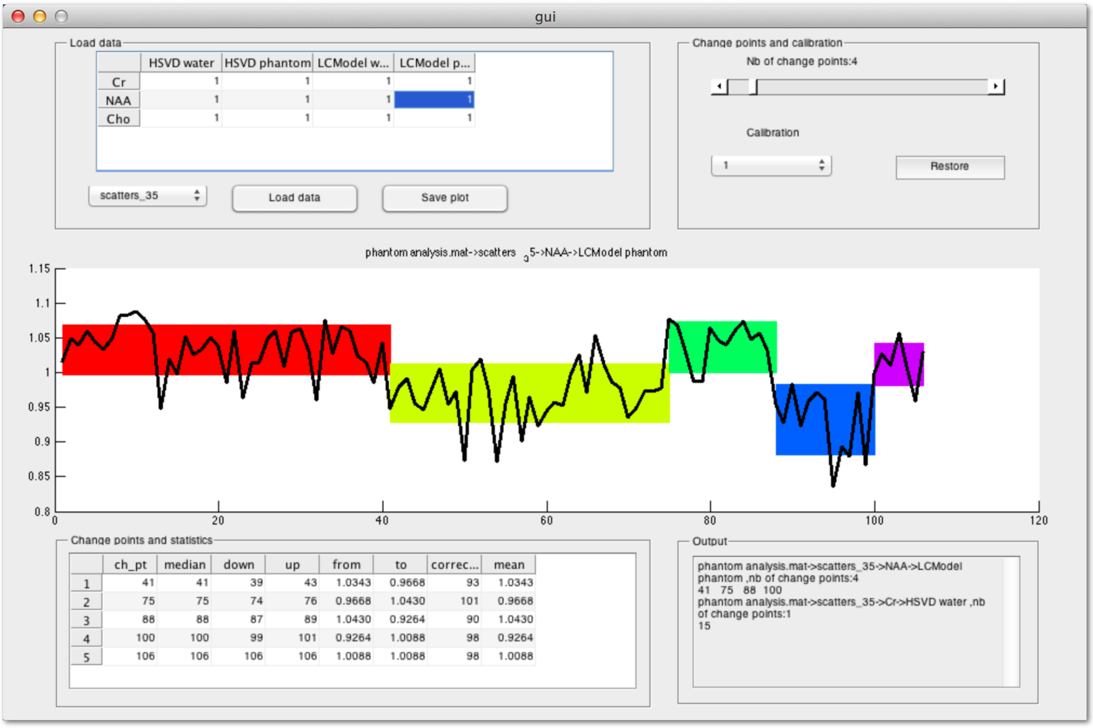

The preview of data content in .xls file is similar but data is stored only in one single row.

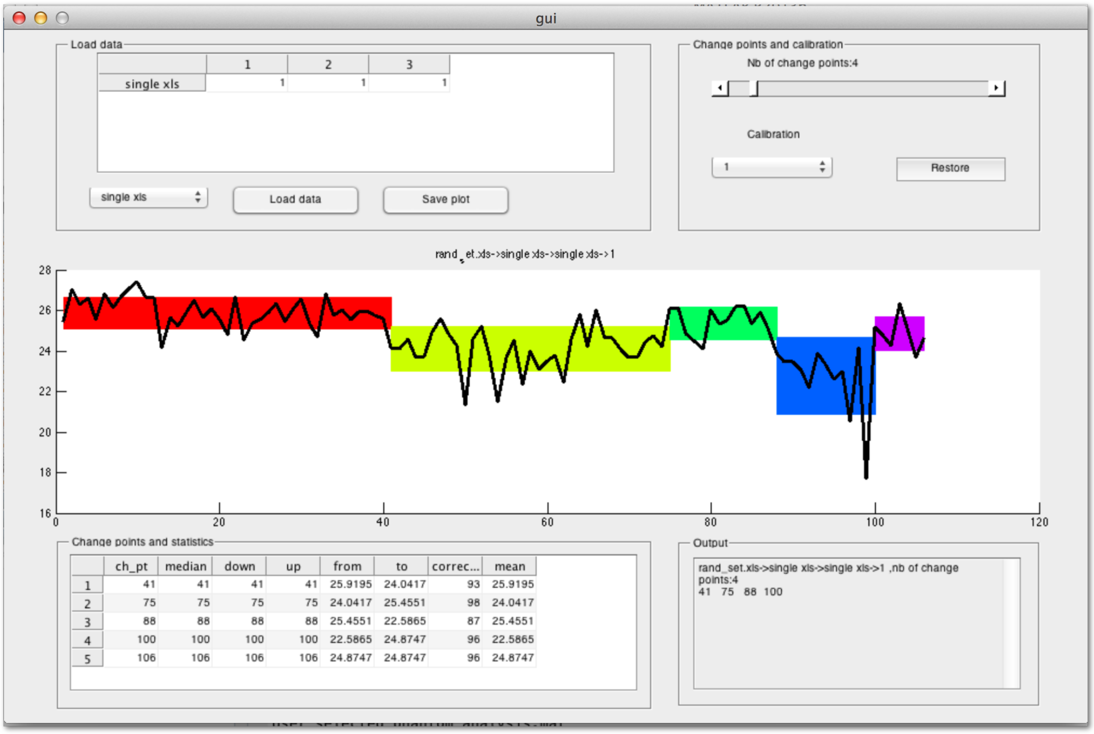

5. Choosing data.

It was assumed that the workspace saved in .mat file may consist of many cell arrays. In order to choose another cell array the user has to indicate it in the popup menu. Data stored in .xls file will have only one single row (with many time series placed in columns).

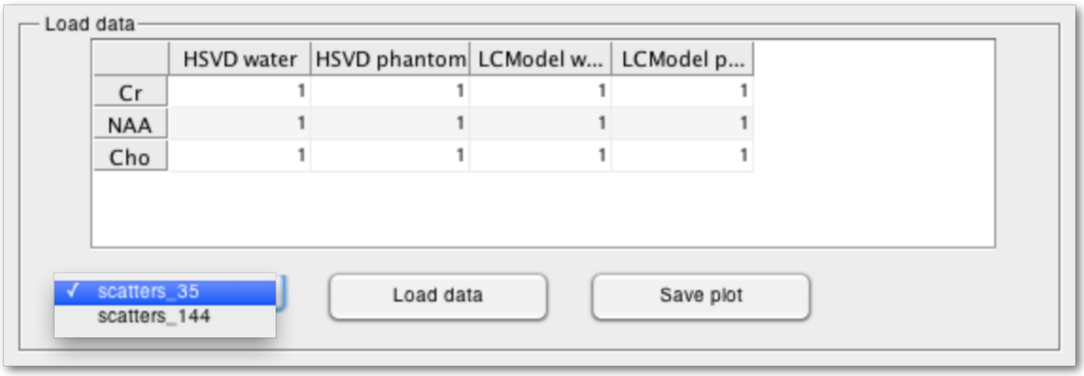

6. Load data and save results.

The Load .mat button enables to load another .mat, whereas the Save plot button saves the results.

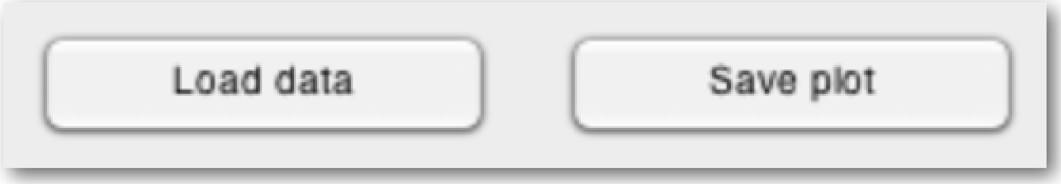

7. Change points detection and calibration.

The GUI allows an automatic detection of change points. However, the users may also define the number of change points manually. With the Calibration button it is possible to define the time interval used in the denominator of the formula of correction factor calculation (section 9).

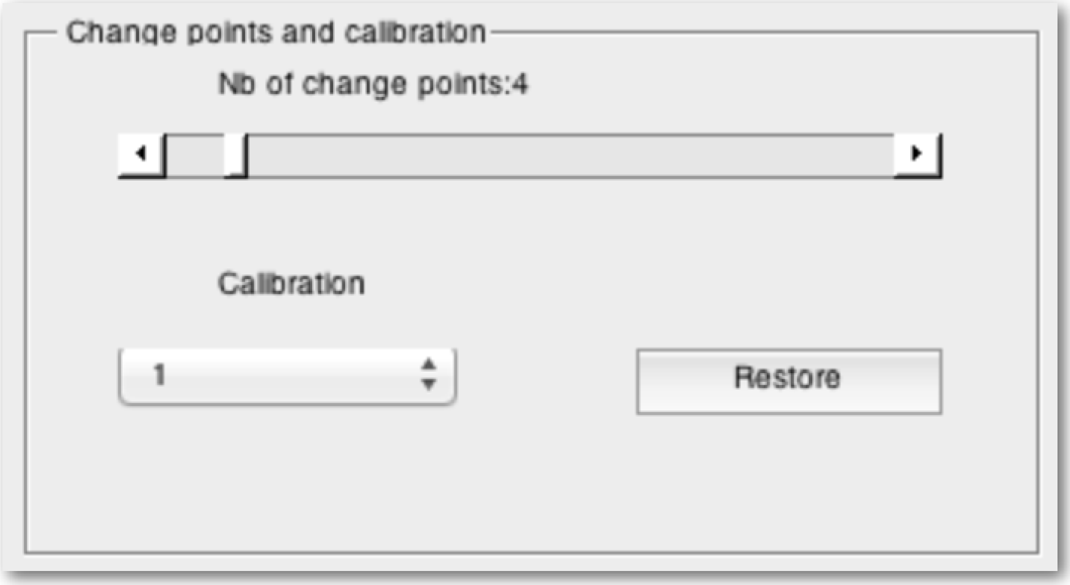

8. Visualization of results.

On the basis of the analysis the monitored time period is divided into several intervals between the calculated change points. These intervals are marked with different colors bands corresponding to mean +/- standard deviation of the signal.

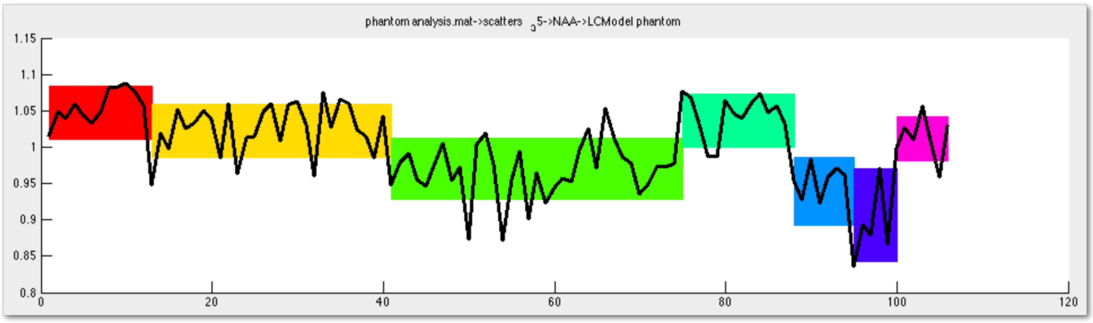

9. Statistics of results.

The numerical results are shown in a table located in the bottom left corner of the box. The table presents: change points (ch_pt), medians, lower and upper confidence intervals (down and up), magnitude of the signal change (from/to) expressed as mean signals in the time intervals before and after the change point, correction factor computed as: (mean signal in the time interval after the change point /mean signal in the first interval)*100%, mean signal in the time period before the change point.

Change points and statistics

	ch_pt	median	down	up	from	to	correc...	mean
1	41	41	39	43	1.0343	0.9668	93	1.0343
2	75	75	74	76	0.9668	1.0430	101	0.9668
3	88	88	87	89	1.0430	0.9264	90	1.0430
4	100	100	99	101	0.9264	1.0088	98	0.9264
5	106	106	106	106	1.0088	1.0088	98	1.0088

The same statistics for 3rd interval treated as reference (100%) can be seen below.

Change points and statistics

	ch_pt	median	down	up	from	to	correc...	mean
1	41	41	38	44	0.9917	0.9269	99	1.0343
2	75	75	73	77	0.9269	1	93	0.9668
3	88	88	87	89	1	0.8883	100	1.0430
4	100	100	99	101	0.8883	0.9672	89	0.9264
5	106	106	106	106	0.9672	0.9672	97	1.0088

10. History window.

The history of all steps done in the GUI during the procedure can be found in the bottom-right corner.

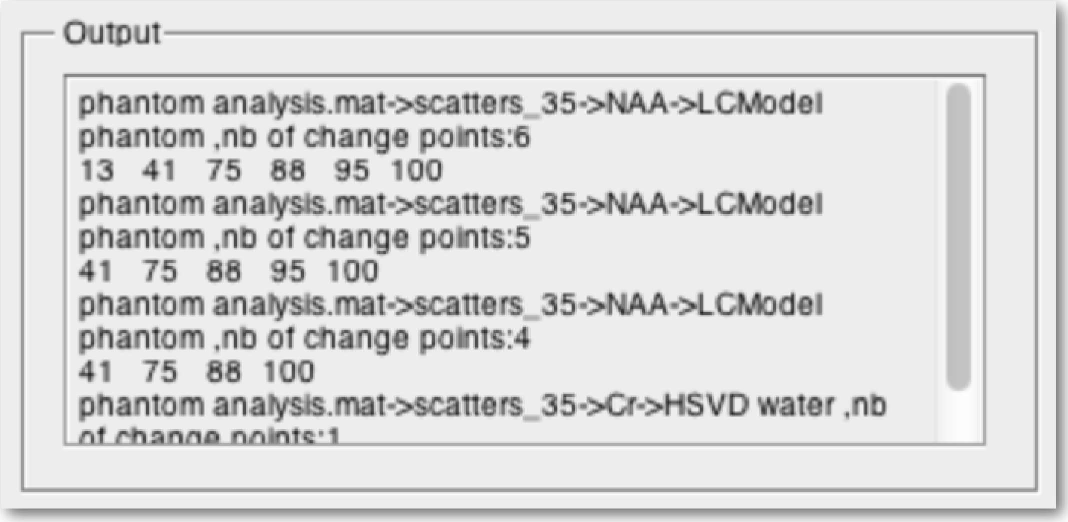

11. Possible problems.

If data format is incorrect all fields of the GUI will be empty showing only 'no data' signature and the preview window will show a flat line. In such case the data format should be corrected.

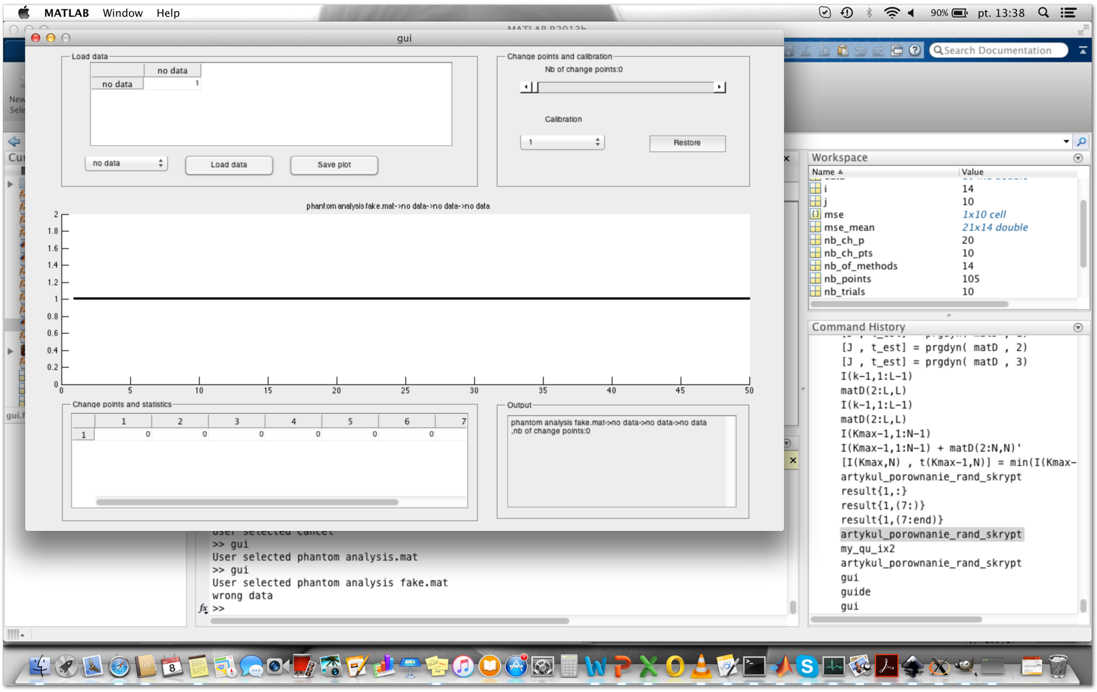

Copyright © All Rights Reserved.
