# Supplementary material for: Quality Control Procedure Based on Partitioning of NMR Time Series
Source: Sensors (Basel). 2018 Mar 6;18(3):792. doi: 10.3390/s18030792 (PMC5877107; doi:10.3390/s18030792)
Supplement: Supplementary file 1 [file sensors-18-00792-s001.zip › Supplementary Materials/manual/3 Matlab scripts.pdf]

Tool for analysis of time series of NMR data

Matlab scripts

1. All scripts and functions.
2. Script demo.

1. List of scripts and functions that are used inside each of them.

dyn_pr_split - determination of change points.

compute_nb_cp - computes number of change points in time series data.
→ dyn_pr_split - determination of change points.

gen_rand - generates random time series, distorted by white noise, which is based on randomly generated change points.

gen_ar - Computes confidence statistics of correct assignment of change points basing on generated ar models. Additionally it illustrates results basing on few plots.
→ compute_ar - generates ar model with given noise in interval of time series either for constant parameters or generated by Matlab functions.
→ gen_rect - computes coordinates of rectangle which illustrates change in interval of given time series. Coordinates are based on mean and standard deviation of given interval.
→ dyn_pr_split - determination of change points.

comp_two - compares two sets of time series with given number of change points and presents results on plot.
→ gen_ar - Computes confidence statistics of correct assignment of change points basing on generated ar models. Additionally it illustrates results basing on few plots.

check_change_points - generates different data and checks reproducibility of change points.
→ gen_rand - generates random time series, distorted by white noise, which is based on randomly generated change points.
→ gen_ar - Computes confidence statistics of correct assignment of change points basing on generated ar models. Additionally it illustrates results basing on few plots.

2. Script demo demonstrates possible usage of software detecting changes in time series according to one of possible choice value:

1. Generate random data and compute confidence statistics.
2. Compare results of change point analysis for two different time series.
3. Generate different data and check reproducibility of change points.

Copyright © All Rights Reserved.
