# Supplementary material for: Quality Control Procedure Based on Partitioning of NMR Time Series
Source: Sensors (Basel). 2018 Mar 6;18(3):792. doi: 10.3390/s18030792 (PMC5877107; doi:10.3390/s18030792)
Supplement: Supplementary file 1 [file sensors-18-00792-s001.zip › Supplementary Materials/manual/5 Contact.pdf]

Tool for analysis of time series of NMR data

About project	GUI	Matlab scripts	Download	Contact

Authors

If you use this software you should cite the following in any resulting publication:
[1] Michał Staniszewski, Agnieszka Skorupa, Łukasz Boguszewicz, Maria Sokół and Andrzej Polański. Quality Control Procedure Based on Partitioning of NMR Time Series.

Contact: Michal.Staniszewski@polsl.pl

Copyright © All Rights Reserved.
