# Supplementary material for: Quality Control Procedure Based on Partitioning of NMR Time Series
Source: Sensors (Basel). 2018 Mar 6;18(3):792. doi: 10.3390/s18030792 (PMC5877107; doi:10.3390/s18030792)
Supplement: Supplementary file 1 [file sensors-18-00792-s001.zip › Supplementary Materials/manual/methods/check_change_points.html]

Function check\_change\_points 

# Function check\_change\_points

Generates different data and checks reproducibility of change points.

## Contents

- Input
- Output
- Copyrights

## Input

- data\_points - number of data points from which time series is built
- nb\_ch\_p - number of expected change points that are to be generated
- nb\_repeat - number of trials of random data generation
- snr - Signal to Noise Ratio in dB included in white noise

## Output

- members - correctness of assignment of change points
- means - mean assignment of change points

## Copyrights

(C) All rights reserved

The code may be used free of charge for non-commercial and educational purposes, the only requirement is that this text is preserved within the derivative work. For any other purpose you must contact the authors for permission. This code may not be redistributed without written permission from the authors.

ABOUT: This software implements our approach to detect changes in multi-variate time series

IMPORTANT: If you use this software you should cite the following in any resulting publication:   
[1] Michal Staniszewski, Agnieszka Skorupa, Lukasz Boguszewicz, Maria Sokol and Andrzej Polanski. Quality Control Procedure Based on Partitioning of NMR Time Series.

```
function [members,means]=check_change_points(data_points,nb_repeat,nb_ch_p,snr)

    %generate set of random data
    [data,ch_p] = gen_rand(data_points,nb_ch_p,nb_repeat,snr);
    members = zeros(nb_repeat,nb_ch_p);
    %iterate over number of repeats
    for i = 1:nb_repeat
%         disp(i)
        %generate confidence intervals of found change points
        [conf_int,~,~] = gen_ar(data(:,i),100,nb_ch_p,0,0,0);
        check = [];
        %built check data from change points and its confidence intervals
        for j=1:size(conf_int,1)
            check = [check conf_int(j,3):conf_int(j,4)];
        end
        members(i,:) = ismember(ch_p(2:end-1),check);
    end
    members(i+1,:) = sum(members,1)/nb_repeat*100;
    means = mean(members(i+1,:));
end
```

```
ans =

     1     1     0     0     1
     0     0     0     1     0
     1     1     0     0     0
     0     1     0     0     0
     0     1     0     1     1
    40    80     0    40    40
```

Published with MATLAB® R2013b
