# Supplementary material for: Quality Control Procedure Based on Partitioning of NMR Time Series
Source: Sensors (Basel). 2018 Mar 6;18(3):792. doi: 10.3390/s18030792 (PMC5877107; doi:10.3390/s18030792)
Supplement: Supplementary file 1 [file sensors-18-00792-s001.zip › Supplementary Materials/manual/methods/compute_ar.html]

Function compute\_ar 

# Function compute\_ar

Generates ar model with given noise in interval of time series either for constant parameters or generated by Matlab functions.

## Contents

- Input
- Output
- Copyrights

## Input

- interval - chosen intrval of time series
- noise - randomly generated noise
- arcoeffs - experimentaly chosen ar parameters
- matlab\_flag - choice of experimental parameters (if 1) or generated by matlab (if 0)

## Output

- x - given interval with randomly generated ar model

## Copyrights

(C) All rights reserved.

The code may be used free of charge for non-commercial and educational purposes, the only requirement is that this text is preserved within the derivative work. For any other purpose you must contact the authors for permission. This code may not be redistributed without written permission from the authors.

ABOUT: This software implements our approach to detect changes in multi-variate time series

IMPORTANT: If you use this software you should cite the following in any resulting publication:   
[1] Michal Staniszewski, Agnieszka Skorupa, Lukasz Boguszewicz, Maria Sokol and Andrzej Polanski. Quality Control Procedure Based on Partitioning of NMR Time Series.

```
function [x]=compute_ar(interval,noise,arcoeffs,matlab_flag)
    N = size(interval,1);
    if matlab_flag
        p = size(arcoeffs,2) - 1;
        randnoise = randn(1,N);
        x=zeros(1,N);
        for k=1:p
            x(k) = x(k) + noise * randnoise(1,k);
        end

        for k=(p+1):N
            for i=1:p
                x(k) = x(k)+arcoeffs(i+1)*x(k-p);
            end
            x(k) = x(k) + noise * randnoise(1,k);
        end
        x = x';
    else
        randnoise = randn(N,1);
        x = filter(1,arcoeffs,noise * randnoise(:,1));
    end
end
```

```
Error using compute_ar (line 29)
Not enough input arguments.
```

Published with MATLAB® R2013b
