# Supplementary material for: Quality Control Procedure Based on Partitioning of NMR Time Series
Source: Sensors (Basel). 2018 Mar 6;18(3):792. doi: 10.3390/s18030792 (PMC5877107; doi:10.3390/s18030792)
Supplement: Supplementary file 1 [file sensors-18-00792-s001.zip › Supplementary Materials/manual/methods/compute_nb_cp.html]

Function compute\_nb\_cp 

# Function compute\_nb\_cp

Computes number of change points in time series data.

## Contents

- Input
- Output
- Copyrights

## Input

- data - the vector containing times series
- plot\_flag - if set to 1, plot all bic values

## Output

- nb\_change\_points - number of change points

## Copyrights

(C) All rights reserved.

The code may be used free of charge for non-commercial and educational purposes, the only requirement is that this text is preserved within the derivative work. For any other purpose you must contact the authors for permission. This code may not be redistributed without written permission from the authors.

ABOUT: This software implements our approach to detect changes in multi-variate time series

IMPORTANT: If you use this software you should cite the following in any resulting publication:   
[1] Michal Staniszewski, Agnieszka Skorupa, Lukasz Boguszewicz, Maria Sokol and Andrzej Polanski. Quality Control Procedure Based on Partitioning of NMR Time Series.

```
function [nb_change_points]=compute_nb_cp(data,plot_flag)

    n = size(data,1);
    cnt = 10; %nb of possible change points
    bic = zeros(cnt,1);
    mse = zeros(cnt,1);
    segments = zeros(size(data,1),cnt);
    wait = waitbar(0,'Calculating number of change points...');
    [~,ch_p] = dyn_pr_split(data,cnt);
    %iterate over number of maximum change points
    for i=1:(cnt)
        waitbar(i / cnt)
        change_points = ch_p(i,1:i);
        if change_points(i) == size(data,1)
        	break
        else
        	change_points = [1; change_points'; size(data,1)];
        end

        means = zeros(size(data,1),1);
        for j=1:i
            first = data((change_points(j)):(change_points(j+1)+1));
            u = ones(size(first));
            means((change_points(j)):(change_points(j+1)+1)) = u*mean(first)';
        end
        first = data((change_points(j+1)):(change_points(j+2)));
        u = ones(size(first));
        means((change_points(j+1)):(change_points(j+2))) = u*mean(first)';
        segments(:,i) = means;
        n = size(data,1);
        if mod(n,2)~=0
            data(n+1)=(data(n-1)+data(n))/2;
            var=sum((data(1:2:(n))-data(2:2:(n+1))).^2)/(n+1);
            data = data(1:n);
        else
            var=sum((data(1:2:(n-1))-data(2:2:n)).^2)/n;
        end
        %calculate bic
        mse(i) = sum(abs(data./means - 1));
        bic(i)=log(mse(i))*n+i*log(n);

    end
    %find minimum of bic
    bic(2:cnt+1) = bic;
    mse(1) = sum(abs(data - 1));
    bic(1)=log(mse(1))*n;
    [~,nb_change_points] = min(bic);
    nb_change_points = nb_change_points-1;
    close(wait)
    %plot bic
    if plot_flag
        figure
        plot(0:cnt,bic,'b')
    end

end
```

```
ans =

     4
```

Published with MATLAB® R2013b
