# Supplementary material for: Quality Control Procedure Based on Partitioning of NMR Time Series
Source: Sensors (Basel). 2018 Mar 6;18(3):792. doi: 10.3390/s18030792 (PMC5877107; doi:10.3390/s18030792)
Supplement: Supplementary file 1 [file sensors-18-00792-s001.zip › Supplementary Materials/manual/methods/comp_two.html]

Function comp\_two 

# Function comp\_two

Compares two sets of time series with given number of change points and presents results on plot.

## Contents

- Input
- Output
- Copyrights

## Input

- data1 - the first vector containing time series
- data2 - the second vector containing time series
- nb\_chpt1 - the number of change points in the first time series data1
- nb\_chpt2 - the number of change points in the second time series data2

## Output

results are presented in the form of plot

## Copyrights

(C) All rights reserved.

The code may be used free of charge for non-commercial and educational purposes, the only requirement is that this text is preserved within the derivative work. For any other purpose you must contact the authors for permission. This code may not be redistributed without written permission from the authors.

ABOUT: This software implements our approach to detect changes in multi-variate time series

IMPORTANT: If you use this software you should cite the following in any resulting publication:   
[1] Michal Staniszewski, Agnieszka Skorupa, Lukasz Boguszewicz, Maria Sokol and Andrzej Polanski. Quality Control Procedure Based on Partitioning of NMR Time Series.

```
function []=comp_two(data1,data2,nb_chpt1,nb_chpt2)

    [ci_1,~,rect_1] = gen_ar(data1,100,nb_chpt1,0,0,0);
    [ci_2,~,rect_2] = gen_ar(data2,100,nb_chpt2,0,0,0);
    y1 = max(max(data1),max(data2))*1.01;
    y2 = y1*1.01;
    figure
    hold on
    for j=1:1:nb_chpt1
        fill(rect_1(j,1).x,rect_1(j,1).y, 'b','EdgeColor','b','FaceAlpha', 0.1)
        plot(ci_1(j,3):ci_1(j,4),ones(1,ci_1(j,4)-ci_1(j,3)+1)*y1,'.','linewidth',3,'color','b')
    end
    fill(rect_1(j+1,1).x,rect_1(j+1,1).y, 'b','EdgeColor','b','FaceAlpha', 0.1)
    for j=1:1:nb_chpt2
        fill(rect_2(j,1).x,rect_2(j,1).y, 'r','EdgeColor','r','FaceAlpha', 0.1)
        plot(ci_2(j,3):ci_2(j,4),ones(1,ci_2(j,4)-ci_2(j,3)+1)*y2,'.','linewidth',3,'color','r')
    end
    fill(rect_2(j+1,1).x,rect_2(j+1,1).y, 'r','EdgeColor','r','FaceAlpha', 0.1)
    plot(data1,'linewidth',3,'color','b')
    plot(data2,'linewidth',3,'color','r')
    print('cmp2','-dpng')
end
```

Published with MATLAB® R2013b
