# Supplementary material for: Quality Control Procedure Based on Partitioning of NMR Time Series
Source: Sensors (Basel). 2018 Mar 6;18(3):792. doi: 10.3390/s18030792 (PMC5877107; doi:10.3390/s18030792)
Supplement: Supplementary file 1 [file sensors-18-00792-s001.zip › Supplementary Materials/manual/methods/demo_example.html]

Demo example 

# Demo example

Demonstrates possible usage of software detecting changes in multi-variate time series

## Contents

- Choice
- Copyrights
- Choice 1
- Choice 2
- Choice 3

## Choice

1. generate random data and compute confidence statistics
2. compare results of change point analysis for two different time series
3. generate different data and check reproducibility of change points

## Copyrights

(C) All rights reserved.

The code may be used free of charge for non-commercial and educational purposes, the only requirement is that this text is preserved within the derivative work. For any other purpose you must contact the authors for permission. This code may not be redistributed without written permission from the authors.

ABOUT: This software implements our approach to detect changes in multi-variate time series

IMPORTANT: If you use this software you should cite the following in any resulting publication:   
[1] Michal Staniszewski, Agnieszka Skorupa, Lukasz Boguszewicz, Maria Sokol and Andrzej Polanski. Quality Control Procedure Based on Partitioning of NMR Time Series.

## Choice 1

```
        nb_data = 100; %number of data points
        nb_ch_pts = 5; %number of change points
        snr = 30; %value of snr
        [data,~]=gen_rand(nb_data,nb_ch_pts,1,snr); %generate random data
        boot_max = 100; %number of repeat for confidence interval
        %nb_change_points = compute_nb_cp(data,0); %compute number of change points
        nb_change_points = 5; %choose manual number of change points
        plot_flag = 1;
        matlab_flag = 0;
        plot_full = 1;
        [conf_int,pkt1,rect] = gen_ar(data,boot_max,nb_change_points,plot_flag,matlab_flag,plot_full);
```

  

## Choice 2

```
        nb_data = 100; %number of data points
        nb_ch_pts = 5; %number of change points
        nb_repeat = 2; %number of generated random data (time series)
        snr = 30; %value of snr
        [data,~]=gen_rand(nb_data,nb_ch_pts,nb_repeat,snr); %generate random data
        nb_ch_pts_1 = nb_ch_pts; %choose manual number of change points
        nb_ch_pts_2 = nb_ch_pts; %choose manual number of change points
        % nb_ch_pts_1 = compute_nb_cp(data(:,1),0); %compute number of change points
        % nb_ch_pts_2 = compute_nb_cp(data(:,2),0); %compute number of change points
        comp_two(data(:,1),data(:,2),nb_ch_pts_1,nb_ch_pts_2);
```

## Choice 3

```
        nb_data = 100; %number of data points
        nb_ch_pts = 5; %number of change points
        nb_repeat = 10; %number of generated random data (time series)
        snr = 30; %value of snr
        [members,means]=check_change_points(nb_data,nb_ch_pts,nb_repeat,snr);
```

Published with MATLAB® R2013b
