# Supplementary material for: Quality Control Procedure Based on Partitioning of NMR Time Series
Source: Sensors (Basel). 2018 Mar 6;18(3):792. doi: 10.3390/s18030792 (PMC5877107; doi:10.3390/s18030792)
Supplement: Supplementary file 1 [file sensors-18-00792-s001.zip › Supplementary Materials/manual/methods/dyn_pr_split.html]

Function dyn\_pr\_split 

# Function dyn\_pr\_split

Calculates change points by use of dynamic programming

## Contents

- Input
- Output
- Copyrights

## Input

- data - time series
- K\_gr - maximum number of change points (intervals)

## Output

- Q - for K\_gr
- opt\_part - matrix of change points from 1 to K\_gr

## Copyrights

(C) All rights reserved.

The code may be used free of charge for non-commercial and educational purposes, the only requirement is that this text is preserved within the derivative work. For any other purpose you must contact the authors for permission. This code may not be redistributed without written permission from the authors.

ABOUT: This software implements our approach to detect changes in multi-variate time series

IMPORTANT: If you use this software you should cite the following in any resulting publication:   
[1] Michal Staniszewski, Agnieszka Skorupa, Lukasz Boguszewicz, Maria Sokol and Andrzej Polanski. Quality Control Procedure Based on Partitioning of NMR Time Series.

```
function [Q,opt_part]=dyn_pr_split(data,K_gr)
    % initialize
    Q=zeros(1,K_gr);
    N=length(data);
    p_opt_idx=zeros(1,N);
    p_aux=zeros(1,N);
    opt_pals=zeros(K_gr,N);
    for kk=1:N;
        p_opt_idx(kk)=my_qu_ix(data(kk:N));
    end

    % iterate
    for kster=1:K_gr
        kster;
       for kk=1:N-kster
           for jj=kk+1:N-kster+1
               p_aux(jj)= my_qu_ix(data(kk:jj-1))+p_opt_idx(jj);
           end
           [mm,ix]=min(p_aux(kk+1:N-kster+1));
           p_opt_idx(kk)=mm;
           opt_pals(kster,kk)=kk+ix(1);
       end
       Q(kster)=p_opt_idx(1);
    end

    % restore optimal decisions
    opt_part=zeros(K_gr,K_gr);
    for i=1:1:K_gr
        opt_part(i,1)=opt_pals(i,1);
        for kster=i-1:-1:1
           opt_part(i,i-kster+1)=opt_pals(kster,opt_part(i,i-kster));
        end
    end
end
```

```
Q =

  Columns 1 through 7

    3.7989    3.6487    3.1689    2.9347    2.7439    2.6547    2.5491

  Columns 8 through 10

    2.4688    2.4079    2.3300


opt_part =

    41     0     0     0     0     0     0     0     0     0
    41   100     0     0     0     0     0     0     0     0
    41    75    88     0     0     0     0     0     0     0
    41    75    88   100     0     0     0     0     0     0
    41    75    88    95   100     0     0     0     0     0
    13    41    75    88    95   100     0     0     0     0
     8    12    41    75    88    95   100     0     0     0
     8    13    14    41    75    88    95   100     0     0
     8    13    14    41    63    75    88    95   100     0
     8    13    14    41    75    78    80    88    95   100
```

Published with MATLAB® R2013b
