# Supplementary material for: Quality Control Procedure Based on Partitioning of NMR Time Series
Source: Sensors (Basel). 2018 Mar 6;18(3):792. doi: 10.3390/s18030792 (PMC5877107; doi:10.3390/s18030792)
Supplement: Supplementary file 1 [file sensors-18-00792-s001.zip › Supplementary Materials/manual/methods/gen_ar.html]

Function gen\_ar 

# Function gen\_ar

Computes confidence statistics of correct assignment of change points basing on generated ar models. Additionally it illustrates results basing on few plots.

## Contents

- Input
- Output
- Copyrights

## Input

- data - the vector containing times series
- boot\_max - number of repeats in order to calculate confidence intervals, should be equal to at least 100
- nb\_change\_points - number of change points in data time series
- plot\_flag - if set to 1, plot all ar models on time series in figure(1) and statistics of change points in figure(3)
- matlab\_flag - choice of experimental parameters (if 1) or generated by matlab (if 0)
- plot\_full - plot (if 1) in figure(2) time series with change intervals in different colors

## Output

- conf\_level - consisting of change points and calculated statistics (which is: change point, median, confidence intervals, change in intervals from to with respect to first one, correction to first interval, mean in intervals)
- pkt - all generated results of change points (related to original change points row by row)
- rect - data needed to use fill function of each interval of changes

## Copyrights

(C) All rights reserved.

The code may be used free of charge for non-commercial and educational purposes, the only requirement is that this text is preserved within the derivative work. For any other purpose you must contact the authors for permission. This code may not be redistributed without written permission from the authors.

ABOUT: This software implements our approach to detect changes in multi-variate time series

IMPORTANT: If you use this software you should cite the following in any resulting publication:   
[1] Michal Staniszewski, Agnieszka Skorupa, Lukasz Boguszewicz, Maria Sokol and Andrzej Polanski. Quality Control Procedure Based on Partitioning of NMR Time Series.

```
function [conf_level,pkt,rect]=gen_ar(data,boot_max,nb_change_points,plot_flag,matlab_flag,plot_full)

    [~,change_points] = dyn_pr_split(data,nb_change_points);
    change_points = [1; change_points'; size(data,1)];
    %plot time series with ar models
    if plot_flag
        figure(1)
        plot(data,'linewidth',3)
        hold on
    end
    %plot statistics
    if plot_full
        figure(2)
        hold on
    end
    cc=hsv(nb_change_points+1);
    conf_level = 1;
    %generate random noise
    noise = rand(1,boot_max)*0.2;
    sub_plot_index = 1;
    wait = waitbar(0,'Calculating confidence intervals...');
    %iterate over number of change points
    for j=1:1:nb_change_points
        waitbar(j / nb_change_points)
        first = data((change_points(j)):(change_points(j+1)-1));
        second = data((change_points(j+1)+1):(change_points(j+2)));
        step = min(size(first,1),size(second,1));
        first = first((end-step+1):end);
        second = second(1:step);
        u1 = ones(size(first));
        u2 = ones(size(second));
        mean_first = mean(first);
        mean_second = mean(second);
        coeffs_1 = [1 0.498864831482671 -0.174395698358703 -0.696974642508722 -0.298142800223909];
        coeffs_2 = coeffs_1;
        %iterate over number of repeats, generate ar models
        for i=1:1:boot_max
            x_first=compute_ar(first,noise(i),coeffs_1,matlab_flag)+mean_first;
            x_second=compute_ar(second,noise(i),coeffs_2,matlab_flag)+mean_second;
            interval = [u1*mean_first; data(change_points(j+1)); u2*mean_second];
            interval_x = [x_first; data(change_points(j+1)); x_second];
            [~,pkt(j,i)] = dyn_pr_split(interval_x,1);
            pkt(j,i) = pkt(j,i) + change_points(j+1) - step - 1;
            if plot_flag
                figure(1)
                plot((change_points(j+1)-step):(change_points(j+1)+step), interval,'color',cc(j,:))
                plot((change_points(j+1)-step):(change_points(j+1)+step), interval_x,'color',cc(j,:))
            end
        end
        %compute confidence intervals according to:
        % McGill, R., J. W. Tukey, and W. A. Larsen. "Variations of Boxplots." The American Statistician. Vol. 32, No. 1, 1978, pp. 12-16.
        CI = 1.7*(1.25*iqr(pkt(j,:)))/(1.35*sqrt(length(pkt(j,:))));
        conf_level(j,1) = change_points(j+1);
        conf_level(j,2) = median(pkt(j,:));
        conf_level(j,3:4) = [floor(median(pkt(j,:)) - CI), ceil(median(pkt(j,:)) + CI)];
        if(j==1)
            norm_mean = mean(data((change_points(j)):(change_points(j+1)-1)));
            conf_level(j,5) = norm_mean/norm_mean;
        else
            conf_level(j,5) = mean(data((change_points(j)):(change_points(j+1)-1)))/norm_mean;
        end
        conf_level(j,6) = mean(data((change_points(j+1)):(change_points(j+2)-1)))/norm_mean;
        conf_level(j,7) = round((conf_level(j,6)/conf_level(1,5)) *100);
        conf_level(j,8) = mean_first;
        if plot_flag
            figure(3)
            subplot(nb_change_points,2,sub_plot_index)
            hist(pkt(j,:))
            subplot(nb_change_points,2,sub_plot_index + 1)
            ksdensity(pkt(j,:))
            sub_plot_index = sub_plot_index + 2;
        end
        [rect(j,1).x,rect(j,1).y]=gen_rect(data,change_points(j),change_points(j+1));
        if plot_full
            figure(2)
            fill(rect(j,1).x,rect(j,1).y, cc(j,:),'EdgeColor',cc(j,:))
        end
    end
    [rect(j+1,1).x,rect(j+1,1).y]=gen_rect(data,change_points(j+1),change_points(j+2));
    if plot_full
        fill(rect(j+1,1).x,rect(j+1,1).y, cc(j+1,:),'EdgeColor',cc(j+1,:))
        plot(data,'linewidth',3,'color','k')
    end
    close(wait)
end
```

  

Published with MATLAB® R2013b
