# Supplementary material for: Quality Control Procedure Based on Partitioning of NMR Time Series
Source: Sensors (Basel). 2018 Mar 6;18(3):792. doi: 10.3390/s18030792 (PMC5877107; doi:10.3390/s18030792)
Supplement: Supplementary file 1 [file sensors-18-00792-s001.zip › Supplementary Materials/manual/methods/gen_rand.html]

Function gen\_rand 

# Function gen\_rand

Generates random time series, distorted by white noise, which is based on randomly generated change points

## Contents

- Input
- Output
- Copyrights

## Input

- nb\_data - number of data points from which time series is built
- nb\_ch\_pts - number of expected change points that are to be generated
- nb\_repeat - number of trials of random data generation
- snr - Signal to Noise Ratio in dB included in white noise

## Output

- result - generated random time series with random change points
- change\_points - generated randomly change points used as a reference

## Copyrights

(C) All rights reserved.

The code may be used free of charge for non-commercial and educational purposes, the only requirement is that this text is preserved within the derivative work. For any other purpose you must contact the authors for permission. This code may not be redistributed without written permission from the authors.

ABOUT: This software implements our approach to detect changes in multi-variate time series

IMPORTANT: If you use this software you should cite the following in any resulting publication:   
[1] Michal Staniszewski, Agnieszka Skorupa, Lukasz Boguszewicz, Maria Sokol and Andrzej Polanski. Quality Control Procedure Based on Partitioning of NMR Time Series.

```
function [result,change_points]=gen_rand(nb_data,nb_ch_pts,nb_repeat,snr)
    offset = 10; %minimal distance between two change points
    change_points = sort(randperm(nb_data-offset, nb_ch_pts)); %random change points
    change_points = change_points(change_points>offset);
    while size(change_points,2)<nb_ch_pts %ensuring given offset
        change_points = sort(randperm(nb_data-offset, nb_ch_pts)); %random change points
        change_points = change_points(change_points>offset);
    end
    change_points = [1 change_points nb_data];
    result=zeros(nb_data-1,nb_repeat);
    segments = rand(nb_repeat,nb_ch_pts+1); %random values of mean
    %iterate over number of repeats
    for i=1:nb_repeat
        data = [];
        %iterate over number of time series
        for j=1:nb_ch_pts+1
            probe = ones(1,change_points(j+1)-change_points(j))*segments(i,j);
            data = [data probe];
        end
        data = awgn(data,snr,'measured')';
        result(:,i) = data;
    end
%     plot(data)
end
```

```
ans =

    0.7873
    0.7670
    0.7528
    0.7720
    0.7484
    0.7553
    0.7475
    0.7368
    0.8054
    0.7429
    0.7518
    0.7682
    0.7363
    0.7301
    0.7764
    0.7520
    0.7245
    0.7393
    0.7463
    0.7452
    0.7499
    0.7322
    0.7488
    0.7308
    0.7382
    0.7522
    0.7736
    0.7746
    0.7227
    0.7745
    0.7262
    0.7597
    0.7560
    0.7491
    0.7376
    0.7230
    0.7283
    0.7532
    0.7448
    0.7581
    0.7397
    0.7143
    0.7102
    0.7246
    0.7574
    0.7407
    0.7548
    0.7272
    0.7372
    0.2511
    0.3936
    0.4182
    0.3870
    0.3835
    0.3921
    0.3745
    0.4085
    0.4163
    0.4119
    0.3919
    0.3669
    0.4235
    0.3997
    0.4182
    0.3994
    0.3953
    0.3672
    0.4099
    0.4406
    0.3915
    0.4072
    0.4073
    0.4317
    0.3731
    0.3794
    0.4072
    0.5306
    0.5268
    0.5392
    0.5533
    0.6135
    0.2997
    0.3451
    0.2586
    0.3124
    0.2853
    0.2999
    0.3081
    0.3115
    0.3231
    0.3174
    0.3130
    0.2926
    0.3041
    0.3151
    0.2787
    0.3220
    0.3211
    0.3164
```

Published with MATLAB® R2013b
