# Supplementary material for: Quality Control Procedure Based on Partitioning of NMR Time Series
Source: Sensors (Basel). 2018 Mar 6;18(3):792. doi: 10.3390/s18030792 (PMC5877107; doi:10.3390/s18030792)
Supplement: Supplementary file 1 [file sensors-18-00792-s001.zip › Supplementary Materials/manual/methods/gen_rect.html]

Function gen\_rect 

# Function gen\_rect

Computes coordinates of rectangle which illustrates change in interval of given time series. Coordinates are based on mean and standard deviation of given interval.

## Contents

- Input
- Output
- Copyrights

## Input

- data - the vector containing times series
- change\_points\_1 - position of first change point being the begin of interval
- change\_points\_2 - - position of second change point being the end of interval

## Output

- x - left and right coordinates for fill function
- y - top and bottom coordinates for fill function

## Copyrights

(C) All rights reserved

The code may be used free of charge for non-commercial and educational purposes, the only requirement is that this text is preserved within the derivative work. For any other purpose you must contact the authors for permission. This code may not be redistributed without written permission from the authors.

ABOUT: This software implements our approach to detect changes in multi-variate time series

IMPORTANT: If you use this software you should cite the following in any resulting publication:   
[1] Michal Staniszewski, Agnieszka Skorupa, Lukasz Boguszewicz, Maria Sokol and Andrzej Polanski. Quality Control Procedure Based on Partitioning of NMR Time Series.

```
function [x,y]=gen_rect(data,change_points_1,change_points_2)
    first = data((change_points_1):(change_points_2));
    left = change_points_1;
    right = change_points_2;
    bottom = mean(first)-std(first);
    top = mean(first)+std(first);
    x = [left left right right];
    y = [bottom top top bottom];
end
```

```
Error using gen_rect (line 29)
Not enough input arguments.
```

Published with MATLAB® R2013b
